# The free radical scavenger Trolox dampens neuronal hyperexcitability, reinstates synaptic plasticity, and improves hypoxia tolerance in a mouse model of Rett syndrome

**DOI:** 10.3389/fncel.2014.00056

**Published:** 2014-02-24

**Authors:** Oliwia A. Janc, Michael Müller

**Affiliations:** ^1^Center for Nanoscale Microscopy and Molecular Physiology of the Brain, Georg-August-Universität GöttingenGöttingen, Germany; ^2^Zentrum für Physiologie und Pathophysiologie, Institut für Neuro- und Sinnesphysiologie, Universitätsmedizin, Georg-August-Universität GöttingenGöttingen, Germany

**Keywords:** oxidative stress, redox signaling, reactive oxygen species (ROS), mitochondrial metabolism, free radical scavenger, neurodevelopmental disorder, synaptic dysfunction, vitamin E

## Abstract

Rett syndrome (RS) causes severe cognitive impairment, loss of speech, epilepsy, and breathing disturbances with intermittent hypoxia. Also mitochondria are affected; a subunit of respiratory complex III is dysregulated, the inner mitochondrial membrane is leaking protons, and brain ATP levels seem reduced. Our recent assessment of mitochondrial function in MeCP2 (methyl-CpG-binding protein 2)-deficient mouse (*Mecp2*^-^^/y^) hippocampus confirmed early metabolic alterations, an increased oxidative burden, and a more vulnerable cellular redox balance. As these changes may contribute to the manifestation of symptoms and disease progression, we now evaluated whether free radical scavengers are capable of improving neuronal and mitochondrial function in RS. Acute hippocampal slices of adult mice were incubated with the vitamin E derivative Trolox for 3–5 h. In *Mecp2*^-^^/y^ slices this treatment dampened neuronal hyperexcitability, improved synaptic short-term plasticity, and fully restored synaptic long-term potentiation (LTP). Furthermore, Trolox specifically attenuated the increased hypoxia susceptibility of *Mecp2*^-^^/y^ slices. Also, the anticonvulsive effects of Trolox were assessed, but the severity of 4-aminopyridine provoked seizure-like discharges was not significantly affected. Adverse side effects of Trolox on mitochondria can be excluded, but clear indications for an improvement of mitochondrial function were not found. Since several ion-channels and neurotransmitter receptors are redox modulated, the mitochondrial alterations and the associated oxidative burden may contribute to the neuronal dysfunction in RS. We confirmed in *Mecp2*^-^^/y^ hippocampus that Trolox dampens neuronal hyperexcitability, reinstates synaptic plasticity, and improves the hypoxia tolerance. Therefore, radical scavengers are promising compounds for the treatment of neuronal dysfunction in RS and deserve further detailed evaluation.

## INTRODUCTION

Rett syndrome (RS) is a neurodevelopmental disorder that almost exclusively affects girls. It arises from spontaneous mutations in an X-chromosomal gene encoding the transcriptional modulator MeCP2 (methyl-CpG-binding protein 2; Hagberg et al., 1983; Amir et al., 1999; Chahrour et al., 2008) and is associated with severe disabilities. In RS, an initially normal development for the first 6–18 months of life is followed by motor dysfunction, cognitive impairment, loss of speech, epilepsy, and severe breathing disturbances with intermittent systemic hypoxia (Hagberg et al., 1983; Julu et al., 2001; Steffenburg et al., 2001; Percy, 2002; Chahrour and Zoghbi, 2007; Stettner et al., 2008; Katz et al., 2009). These symptoms result from an impaired synaptic maturation and plasticity as well as a general dysfunction of MeCP2-deficient neuronal networks. Yet, despite the severity of symptoms, pronounced neurodegeneration does not occur in RS (Armstrong et al., 1995; Guy et al., 2007).

There is substantial evidence that also mitochondria are impaired in RS. Typical morphological alterations of mitochondria are membrane changes, granular inclusions, vacuolizations, and a swollen appearance (Ruch et al., 1989; Eeg-Olofsson et al., 1990; Cornford et al., 1994; Belichenko et al., 2009). Alterations of mitochondrial function include decreased levels of succinate-cytochrome c reductase and cytochrome c oxidase, a proton leak across the inner mitochondrial membrane, and a reduced respiratory capacity (Coker and Melnyk, 1991; Kriaucionis et al., 2006; Gibson et al., 2010; Li et al., 2013). Furthermore, lowered blood serum levels of vitamin E (Formichi et al., 1998) and a reduced activity of the reactive oxygen species (ROS)-detoxifying enzyme superoxide dismutase (SOD) are evident (Sierra et al., 2001). These deficiencies in cellular ROS-scavenging capabilities combined with impaired mitochondrial function could well contribute to the intensified protein- and lipid-oxidation that is detectable in patient blood samples (Sierra et al., 2001; De Felice et al., 2009), and which provided convincing evidence that RS is associated with oxidative stress [see: (De Felice et al., 2012b)].

Following these indications we have recently analyzed mitochondrial function in the hippocampus of male *Mecp2* knock-out mice (*Mecp2*^-^^/y^). In acute tissue slices of adult mice we confirmed an increased basal mitochondrial respiration and less intensely polarized mitochondria. As mitochondrial respiration is already intensified after the 1st postnatal week, these alterations represent early defects in RS that may facilitate disease progression (Groβer et al., 2012). Using the genetically encoded optical redox sensor roGFP1 (Dooley et al., 2004; Hanson et al., 2004; Funke et al., 2011), we also confirmed a more oxidized and more vulnerable cellular redox balance in neonatal *Mecp2*^-^^/y^ hippocampus (Groβer et al., 2012). Furthermore, incubating organotypic slices with the radical scavenger Trolox improved cellular redox conditions, which identifies radical scavenger treatment as a potential pharmacotherapy in RS. This is also supported by a report that a diet rich in ω-3 polyunsaturated fatty acids successfully decreases the severity of the clinical appearance and lowers the levels of various oxidative stress markers in Rett patients (De Felice et al., 2012a). It is therefore tempting to hypothesize that the chronic oxidative stress in RS underlies at least some of the typical symptoms and contributes to disease progression.

In the present study, we therefore evaluated the pharmacotherapeutic potential of the radical scavenger Trolox, a water soluble vitamin E derivative, in RS. Vitamin E and its derivatives prevent the peroxidation of unsaturated lipids in cell membranes and lipoproteins (Wang and Quinn, 1999; Slemmer et al., 2008). Since vitamin E levels are decreased in the blood serum of Rett patients (Formichi et al., 1998), supplementation with vitamin E and/or its derivatives is a logical approach. In detail, we elucidated the potential merit of Trolox in acute hippocampal tissue slices of adult wildtype (WT) and *Mecp2*^-^^/y^ mice. Our focus was on a potential improvement of synaptic function and plasticity, hypoxia tolerance, and mitochondrial function in the tissue of already symptomatic animals. For several of the tested parameters, which are affected in RS, we found an improvement – often to those conditions typical for WT mice. We therefore conclude that radical scavenger treatment is a promising pharmacotherapeutic approach in RS which deserves further detailed analyses.

## MATERIALS AND METHODS

### PREPARATION

As a mouse model for RS, we continued to use mice lacking the *MECP2* gene [B6.129P2(C)-*Mecp*^2tm^^-^^1^^-^^1Bird^ (Guy et al., 2001)]. Heterozygous female mice were obtained from Jackson Laboratories and bred with WT males (C57BL/6J) to generate heterozygous females, hemizygous males, and WT mice of either gender. All experiments were performed on acute tissue slices obtained from adult hemizygous males (*Mecp2*^-^^/y^) around postnatal day 40–50. At this stage, all *Mecp2*^-^^/y^ animals showed characteristic RS symptoms, including a ~40% reduction in body weight, smaller brain size, low motor activity, very frequent hind-limb clasping, obvious breathing disturbances (Guy et al., 2001), as well as frequent seizures during anesthesia. Only male mice were used for the experiments due their earlier and more severe phenotype and in particular to ensure a consistent and complete MeCP2-deficiency in the analyzed brain tissue.

Deeply ether anesthetized mice were decapitated, the brain was rapidly removed from the skull and placed in chilled artificial cerebrospinal fluid (ACSF) for 1–2 min. Acute neocortical/hippocampal tissue slices (400 μm thick transverse slices) were cut from the forebrain using a vibroslicer (Campden Instruments, 752M Vibroslice). The slices were then separated in the sagittal midline and depending on the very type of experiment they were either directly transferred to an interface recording chamber or to a separate submersion-style storage chamber. In any case, slices were left undisturbed for at least 90 min before the experiments were started.

### SOLUTIONS

All chemicals were obtained from Sigma–Aldrich, unless stated otherwise. ACSF was composed of (in mM): 130 NaCl, 3.5 KCl, 1.25 NaH_2_PO_4_, 24 NaHCO_3_, 1.2 CaCl_2_, 1.2 MgSO_4_, and 10 dextrose; it was aerated constantly with 95% O_2_ - 5% CO_2_ (carbogen) to adjust pH to 7.4. The free radical scavenger Trolox [(+/-)-6-hydroxy-2,5,7,8-tetramethylchromane-2-carboxylic acid] and the convulsant 4-aminopyridine (4-AP) were directly added to the ACSF in their final concentrations. Cyanide (CN^-^, sodium salt) was dissolved as an aqueous 1 M stock solution and stored at -20°C; CN^-^ working dilutions were prepared freshly immediately before use. FCCP [carbonyl cyanide 4-(trifluoromethoxy) phenylhydrazone, Tocris] and Rh123 were dissolved in dimethyl sulfoxide (DMSO) as 10 mM and 20 mg/ml stocks, respectively, and stored at 4°C; final DMSO concentrations were <0.05%.

### HYPOXIA PROTOCOL AND ELECTROPHYSIOLOGICAL RECORDINGS

Electrophysiological recordings were performed in an Oslo style interface recording chamber. It was kept at a temperature of either 31-32°C (synaptic function and plasticity) or 35-36°C (hypoxia and seizures), continuously aerated with carbogen (400 ml/min), and perfused with oxygenated ACSF (3-4 ml/min). Severe hypoxia was induced by switching the recording chamber’s gas supply from carbogen to 95% N_2_ - 5% CO_2_ (carbogen aeration of the ACSF was continued), and it triggered hypoxia-induced spreading depression (HSD) -like depolarizations within a few minutes. O_2_ was resubmitted 30 s after the onset of HSD, within that time the extracellular DC potential shift had fully reached its nadir. Extracellular recording electrodes were pulled from thin-walled borosilicate glass (GC150TF-10, Harvard Apparatus) on a horizontal electrode puller (Model P-97, Sutter Instruments). They were filled with ACSF, and their tips were trimmed to a resistance of ~5 MΩ

Field excitatory postsynaptic potentials (fEPSPs) were elicited by 0.1 ms unipolar stimuli (S88 stimulator with PSIU6 stimulus isolation units, Grass Instruments), and delivered via steel microwire electrodes (50 μm diameter, AM-Systems) to the Schaffer collaterals. The resulting orthodromic responses and the extracellular DC potential shifts associated with HSD were measured in *st. radiatum* of the cornu ammonis 1 (CA1) subfield. Seizure-like events (SLEs) were monitored in *st. pyramidale* of the CA3 region. All electrophysiological data were recorded with a locally constructed extracellular DC potential amplifier (Hepp et al., 2005) and sampled using an Axon Instruments Digitizer 1322A and PClamp 9.2 software (Molecular Devices). HSD was sampled at 2.5 kHz, evoked potentials and SLEs were sampled at 20 kHz.

Synaptic plasticity was analyzed by paired-pulse protocols and LTP-inducing protocols. For paired-pulse facilitation (PPF), stimulus intensity was adjusted to obtain half-maximum responses and the inter-stimulus interval was varied in between 25 and 200 ms. LTP was induced in the presence of normal extracellular Ca^2^^+^ concentration (1.2 mM) by applying stimuli of corresponding intensity at a rate of 100 Hz. These stimuli were delivered in three trains of 1 s duration each and separated by 5 min intervals.

### OPTICAL RECORDINGS

Imaging of flavin adenine dinucleotide (FAD) and nicotinamide adenine dinucleotide (NADH) autofluorescence as well as mitochondrial membrane potential (Δψ_m_) was performed on the tissue level, using a computer-controlled digital imaging system. It was composed of a polychromatic xenon-light source (Polychrome II, Till Photonics) and a sensitive CCD camera (Imago QE, PCO Imaging). This camera type is equipped with a 2/3 inch CCD chip (1376×1040 pixels; 6.45 μm × 6.45 μm on chip pixel size), and it exhibits a 62% quantum efficiency at 500 nm.

For the imaging of slices a submersion-style chamber (30-32°C) and a 40x water immersion objective (Zeiss Achroplan, 0.8 NA) were used; the slices were kept in place by a nylon-wired platinum grid. To rate mitochondrial metabolism, FAD and NADH autofluorescence were monitored in a ratiometric approach by alternate excitation at 445 nm (FAD) and 360 nm (NADH); autofluorescence was recorded using a 450 nm beam-splitter and a 510/80 nm bandpass filter (Duchen and Biscoe, 1992; Foster et al., 2006; Gerich et al., 2006). 4×4 pixel binning was applied to increase the detection sensitivity of the CCD camera. Rh123, a marker of Δψ_m_ (Emaus et al., 1986; Duchen, 1999; Foster et al., 2006), was excited at 480 nm and its emission was recorded in 2×2 pixel binning mode, using a 505 nm beam-splitter and a 535/35 nm bandpass filter. Slices were bulk loaded with Rh123 (5 μM, 15 min) in a miniaturized staining chamber (Funke et al., 2007; Groβer et al., 2012). Rh123 was used in quenching mode. Accordingly, mitochondrial depolarization is indicated by an increase in Rh123 fluorescence due to its release from mitochondria into the cytosol. To minimize the risk of phototoxicity that may arise especially from short-wavelength (near-UV) illumination, the imaging experiments – which in part lasted up to 1 h – were performed at low frames rates, acquiring images every 5 s only. Furthermore, the illumination was minimized to those exposure times yielding sufficiently stable CCD camera readings (NADH 70 ms, FAD 40 ms, Rh123 5 ms).

Fluorescence intensities were averaged within defined regions of interest (size ~50 × 50 μm) placed in the middle of *st. radiatum *(see **Figure 4D**), since synaptic function was characterized in this layer as well. The intensity changes observed were referred to pre-treatment baselines; background correction was not performed. As intended by this approach, all optical analyses yield information from a tissue volume rather than single cells and thus represent a mixed signal from neurons and glia. Cellular boundaries or organelle structures could not be identified in the low-magnification epifluorescence recordings of tissue autofluorescence or tissue Rh123 fluorescence.

### STATISTICS

Since the electrophysiological and optical experiments lasted only ~1 h, we used up to six slices from each brain. Nevertheless, to ensure independence of observations, every series of experiments was run on at least four different mice of each genotype. All numerical values are reported as mean ± standard deviation; the number of experiments (n) refers to the number of slices analyzed. Statistical significance of the changes observed was tested by two-tailed, unpaired Student’s *t*-tests and a significance level of *P* = 0.05. In the diagrams, statistically significant changes are indicated by asterisks (^*^P < 0.05; ^**^P < 0.01; ^***^P < 0.001), and the corresponding *P* values are reported in the text.

## RESULTS

To rate the potential merits of a treatment of MeCP2-deficient neuronal networks with free radical scavengers, we preincubated acute hippocampal tissue slices of adult WT and *Mecp2*^-^^/y^ mice with 1 mM Trolox for at least 3 h (range 3–5 h). The effects on synaptic function and synaptic plasticity, neuronal excitability, hypoxia susceptibility as well as mitochondrial function were then assessed in the continued presence of Trolox. To be able to screen for a potential reversal of typical RS symptoms, these analyses were performed at an age, at which male Rett mice already show clear phenotypic symptoms, i.e., around postnatal day p40–50.

### MODULATION OF NEURONAL EXCITABILITY AND SYNAPTIC FUNCTION

**FIGURE 1:**
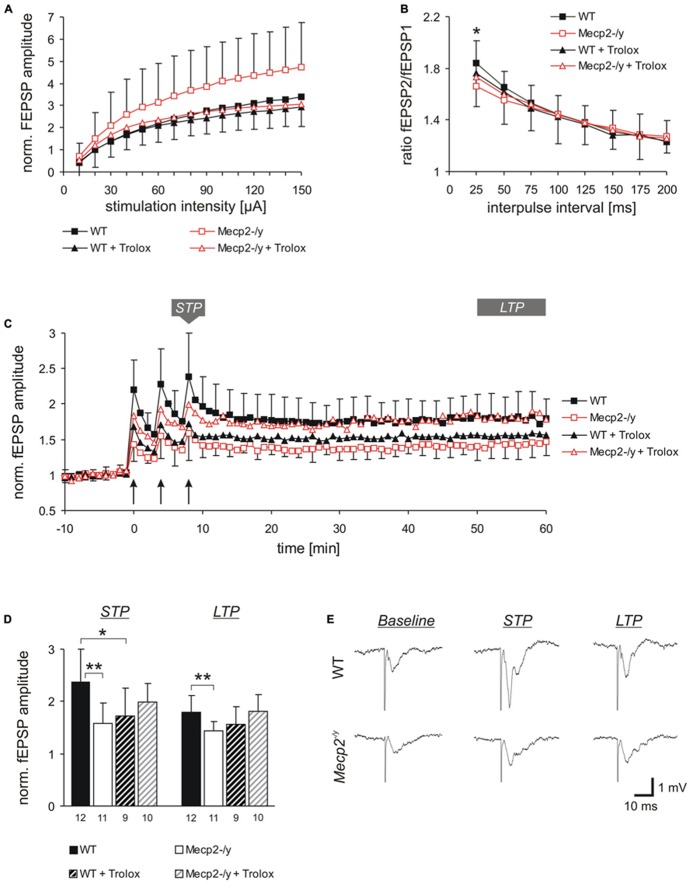
** Trolox dampens neuronal hyperexcitability and reinstates LTP in *Mecp2*^-^^/y^ hippocampus**. **(A)** Input-output curves showing a significantly increased excitability in *Mecp2*^-^^/y^ slices as compared to WT at all stimulation intensities (10–20 μA *P* < 0.05; 30–60 μA *P* < 0.01, 70–150 μA *P* < 0.001). Trolox (1 mM, 3–5h) abolished this genotypic difference. The plotted fEPSP amplitudes are normalized to the fiber volley of the respective slice. Displayed are the averages of 37–50 slices, and error bars represent standard deviations; for clarity they are shown for *Mecp2*^-^^/y^ and *Mecp2*^-^^/y^ plus Trolox only. **(B) **Paired-pulse facilitation (PPF), a measure of short-term plasticity, was less pronounced in *Mecp2*^-^^/y^ than in WT slices for the shortest interpulse-interval tested. Trolox abolished this genotypic difference, but otherwise did not mediate any noticeable effects. Plotted are the averages of 35–52 slices; asterisks indicate statistically significant changes among WT and *Mecp2*^-^^/y^ slices (*P < 0.05). **(C)** STP and LTP were less intense in *Mecp2*^-^^/y^ slices. Trolox improved both types of plasticity in *Mecp2*^-^^/y^ slices and LTP recovered to levels seen in untreated WT slices. In WT, Trolox dampened the extent of LTP to conditions typically found in untreated *Mecp2*^-^^/y^ slices. Averages of 9–12 slices are shown. Error bars are included for every second data point of WT and *Mecp2*^-^^/y^ slices only. LTP was induced by three consecutive trains of 100 Hz stimuli, lasting 1 s each (see arrow marks). **(D)** Comparison of the extent STP and LTP induced in the different groups. The number of slices analyzed is indicated at the bottom of the bars. Asterisks indicate statistically significant changes as compared to WT (**P < 0.01). **(E)** Sample traces of fEPSPs recorded for both genotypes in ACSF under baseline conditions, immediately after the 3rd high-frequency stimulation (STP), and 1 h after inducing potentiation (LTP). Stimulus artifacts are truncated.

Basal synaptic function was rated based on the recording of orthodromically evoked excitatory field potentials (fEPSP) in *st. radiatum* of the CA1 subfield. The fEPSP amplitudes were normalized to the fiber volley (presynaptic compound action potential) to account for differences among the individual slices and variations in electrode positioning. Under control conditions, *Mecp2*^-^^/y^ slices (*n* = 37) showed significantly (~44%) higher fEPSP/fiber volley ratios than WT slices (*n* = 50) at all stimulation intensities tested, which indicates an increased postsynaptic responsiveness and neuronal hyperexcitability (**Figure 1A**). The fiber volley itself and the general shape of the input–output curves did, however, not differ among genotypes; neither could multiple population spikes as a clear sign of pronounced hyperexcitability be observed on a regular basis.

Trolox treatment of slices (1 mM, 3–5 h) abolished the genotypic differences in fEPSP/fiber volley ratios, by specifically decreasing the responses in *Mecp2*^-^^/y^ slices (*n* = 43) to those levels observed in untreated and treated WT slices. Obvious changes in the shape of the input-output curves were not observed upon Trolox treatment (**Figure 1A**). In WT, Trolox did not induce any significant changes in the fEPSP/fiber volley ratio (*n* = 44).

Synaptic plasticity is markedly impaired in Rett mice (Asaka et al., 2006; Moretti et al., 2006; Guy et al., 2007; Fischer et al., 2009). Therefore, we also analyzed the effects of Trolox on various types of synaptic modulation. Synaptic short-term plasticity was assessed as PPF based on twin-pulse stimulation (**Figure 1B**). Stimulation intensity was adjusted to evoke half-maximum response amplitudes and the interpulse-interval was varied between 25 and 200 ms. Whereas this potentiated the amplitude of the 2nd fEPSP in WT slices to 184.0 ± 34.4% (*n* = 52) of control, *Mecp2*^-^^/y^ slices showed a significantly less pronounced fEPSP facilitation to only 165.9 ± 35.7% (*n* = 35; *P* = 0.020) for the shortest interpulse interval tested (25 ms, **Figure 1B**). Trolox treatment abolished this moderate genotypic difference in short-term plasticity at the 25 ms interval, but otherwise did not induce any significant changes in WT (*n* = 47) and *Mecp2*^-^^/y^ slices (*n* = 39).

Furthermore, we assessed the modulation of short-term potentiation (STP) and LTP by Trolox. STP and LTP were induced by high-frequency stimulation (**Figure 1C**). Right after the 3rd stimulus train, fEPSPs were potentiated to 238.2 ± 62.1% (*n* = 12) of their baseline amplitudes in WT slices, but in *Mecp2*^-^^/y^ the extent of STP averaged only 158.7 ± 38.0% (*n* = 11, *P* = 0.001). One hour after LTP induction (range 50–60 min), fEPSPs were still potentiated to 179.4 ± 31.0% in WT slices, but showed a less intense degree of LTP in *Mecp2*^-^^/y^ (143.3 ± 18.5%, *P* = 0.006; **Figures 1C–E**).

Trolox treatment improved the extent of both, STP and LTP in *Mecp2*^-^^/y^ slices. After the 3rd stimulus train fEPSPs were potentiated to 199.3 ± 35.2% and after 1 h they measured 181.1 ± 32.2% (*n* = 10). In WT, a stimulating effect of Trolox was not observed. Instead, the extent of STP slightly declined to 172.4 ± 53.7% (*n* = 9, *P* = 0.020) and LTP showed a tendency of being somewhat less pronounced in the presence of Trolox (155.8 ± 33.2%, *P* = 0.102; **Figures 1C–E**) than in untreated WT slices.

### ANTICONVULSIVE POTENTIAL OF TROLOX

Rett patients show an increased incidence of epileptic seizures (Hagberg et al., 1983; Steffenburg et al., 2001) and increased neuronal excitability is also evident in MeCP2-deficient mice (Medrihan et al., 2008; Fischer et al., 2009; Calfa et al., 2011; McLeod et al., 2013; Toloe et al., 2014). Since the Trolox-mediated decrease in fEPSP/fiber-volley ratio in *Mecp2*^-^^/y^ slices confirms successful dampening of neuronal hyperexcitability, we also tested for potential anticonvulsive effects of this free radical scavenger.

**FIGURE 2:**
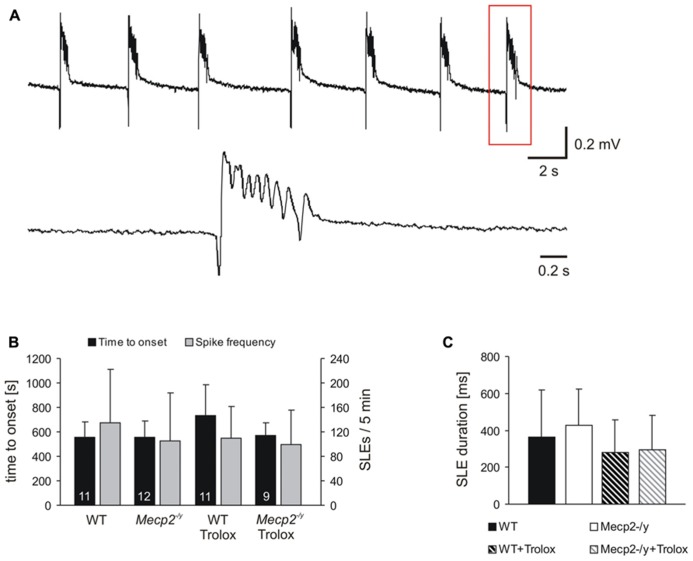
** Trolox only shows a general tendency to dampen seizure susceptibility.** **(A) **Seizure-like discharges (SLEs) recorded from an untreated *Mecp2*^-^^/y^ slice in CA3 *st. pyramidale*. The lower trace shows a SLE (red box) at a stretched time scale. **(B) **Summary of the times to onset and the number of SLEs recorded in the two genotypes. Only in WT Trolox tended to postpone the onset of discharges as compared to untreated slices. The number of slices analyzed is indicated and applies to panel C as well. **(C)** Statistically significant differences in the duration of the individual SLEs among the genotypes were not observed. Trolox only tended to somewhat reduce the duration of the individual discharges in WT and *Mecp2*^-^^/y^ slices.

Seizure activity was provoked by 4-AP (Rutecki et al., 1987), and the resulting SLEs were recorded extracellularly in *st. pyramidale* of the CA3 subfield, as hyperexcitability in Rett mouse**hippocampus arises particularly in this region (Calfa et al., 2011). In about two-thirds of the slices tested, 4-AP (100 μM, 35 min treatment) triggered SLEs which discharged at frequencies of 20–27/min (**Figure 2A**). In WT, SLEs arose within 9.3 ± 2.1 min of 4-AP application and during the last 5 min of treatment, an average number of 135.5 ± 87.3 discharges occurred. The duration of the individual SLEs was quite variable, averaging 362 ± 255 ms (*n* = 11, **Figures 2B,C**). In *Mecp2*^-^^/y^ slices, similar parameters were recorded; SLEs started within 9.2 ± 2.3 min of 4-AP treatment and 105.3 ± 77.8 discharges were registered during the last 5 min; the individual SLEs exhibited an average duration of 429 ± 193 ms (*n* = 12; **Figures 2B,C**). Trolox treatment (1 mM, 3–5 h) showed a solid tendency to postpone the onset of SLEs in WT slices only (*n* = 11, *P* = 0.058); the frequency of discharges was not significantly affected (**Figure 2B**). Also the duration of the individual SLEs only showed a tendency to decrease upon Trolox treatment in both WT (*n* = 11) and *Mecp2*^-^^/y^ slices (*n* = 9), yet the level of significance was not reached (**Figure 2C**).

### TROLOX TREATMENT NORMALIZES THE HYPOXIA SUSCEPTIBILITY

Previously we reported that MeCP2-deficient hippocampus shows an increased susceptibility to hypoxia. As a consequence, the onset of the synchronized response to severe hypoxia – known as HSD – is significantly hastened in adult *Mecp2*^-^^/y^ hippocampal slices. Accordingly, MeCP2-deficient neurons tolerate only a shorter duration of O_2_ shortage and/or chemically induced anoxia before neuronal membrane potentials collapse and neural function ceases (Fischer et al., 2009; Kron and Müller, 2010).

**FIGURE 3:**
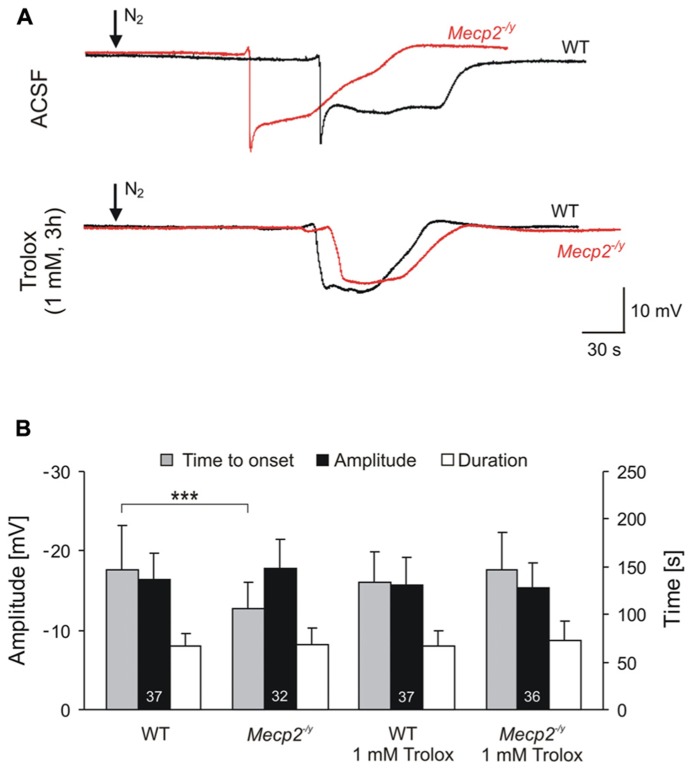
** Trolox improves the hypoxia tolerance of *Mecp2*^-^^/y^ hippocampus**. **(A)** Extracellular DC potential deflections associated with HSD. Note that upon O_2_ withdrawal, HSD occurs earlier in *Mecp2*^-^^/y^ than in WT slices. Incubation of slices with Trolox (1 mM, 3–5 h) selectively postponed HSD onset in *Mecp2*^-^^/y^ slices to values typically observed in WT. The time point of O_2_ withdrawal is indicated by the arrows; oxygenation was restored 30 s upon HSD onset. Recordings were performed in CA1 *st. radiatum*. **(B)** Statistical comparison of the characteristic parameters of the HSD-associated DC potential shift, i.e., amplitude of the negative DC shift (plotted versus the left-hand ordinate in mV) as well as its time to onset and duration (both plotted versus the right-hand ordinate in s). Trolox postponed the onset of HSD in *Mecp2*^-^^/y^ slices, but did not affect the other parameters or its properties in WT. The number of slices analyzed is reported; genotypic differences are indicated by asterisks (***P < 0.001).

For a comparison of hypoxic responses, HSD was induced in WT and *Mecp2*^-^^/y^ slices with and without Trolox treatment. Similar to what was seen earlier, untreated *Mecp2*^-/y^ slices**generated HSD within 1.8 ± 0.5 min upon O_2_ withdrawal (*n* = 32), i.e., 28% earlier than WT slices in which HSD occurred after 2.5 ± 0.8 min of severe hypoxia (*n* = 37, *P* < 0.001; **Figure 3A**). The amplitude and duration of the HSD-associated extracellular DC potential shift did not differ among genotypes (**Figure 3B**). Upon Trolox treatment (1 mM, 3–5 h; Trolox recirculated in the interface chamber) HSD occurred markedly delayed in *Mecp2*^-^^/y^ slices, i.e., after 2.5 ± 0.7 min (*n* = 36, *P* < 0.001), and its time to onset did no longer differ from WT slices. Interestingly, in WT slices, Trolox did not postpone the occurrence of HSD (*n* = 37; **Figure 3**).

### MODULATION OF MITOCHONDRIAL FUNCTION

**FIGURE 4:**
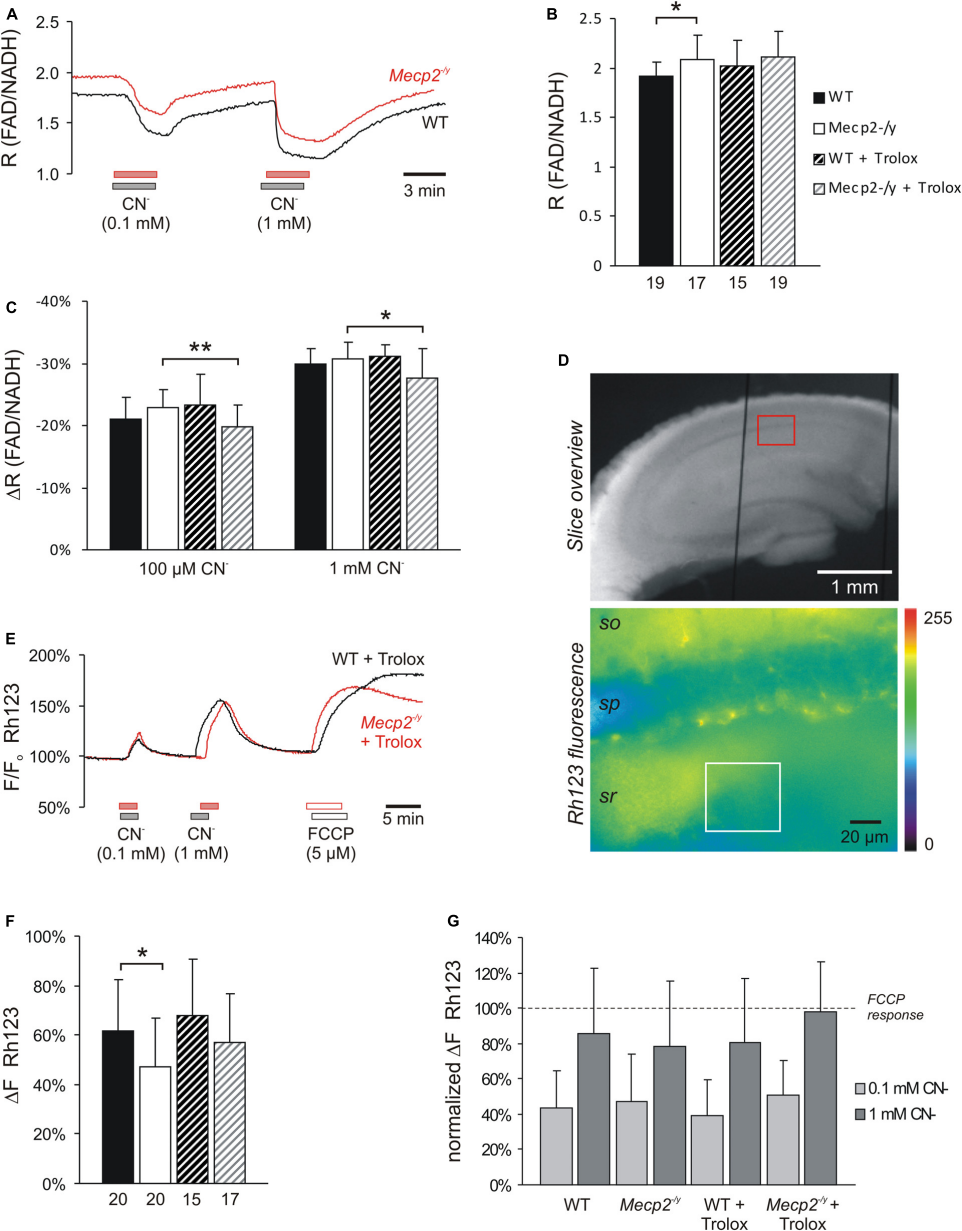
** Trolox only slightly affects mitochondria**. **(A)** Sample traces of the decreases in FAD/NADH ratio induced by CN^-^ in untreated WT and *Mecp2*^-^^/y^ slices. To clarify the baseline difference among genotypes, the traces were superimposed. Gray and red bars indicate the time points and duration of drug application to the WT and *Mecp2*^-^^/y^ slice, respectively. **(B)** The FAD/NADH baseline ratio is moderately increased in *Mecp2*^-^^/y^ slices, indicating intensified mitochondrial respiration. Trolox (1 mM, 3–5 h) did not affect basal respiration. Autofluorescence was analyzed in CA1 *st. radiatum*. Bar shading and patterns are identical for panels **B, C, F** (*P < 0.05). **(C)** Mitochondrial targeting by CN**^-^ (3 min) arrests the respiratory chain, and thereby decreases the FAD/NADH ratio. Trolox slightly dampened these effects of CN^-^ in *Mecp2*^-^^/y^ slices but not in WT (** *P* < 0.01). **(D)** Rh123-labeled slice viewed under white-light illumination and 485 nm excitation. The overview image was taken with a 5x objective, and it also shows two of the vertical-oriented nylon strings immobilizing the slice. The red box indicates the zoomed field of view used for the Rh123 recordings. Fluorescence analyses were performed with a 40x objective and represent an integrated glial/neuronal signal of a tissue volume. Cell boundaries or mitochondrial structures are not identifiable, only the pyramidal cell layer is somewhat less intensely labeled. The white box indicates a typical region of interest analyzed (*so st. oriens; sp st. pyramidale; sr st. radiatum*). **(E)** Superimposed sample traces of the relative Rh123 fluorescence changes (F/F_o_) evoked by CN**^-^ and FCCP in a Trolox-treated WT and *Mecp2*^-^^/y^ slice. Drug treatment of the WT and *Mecp2*^-^^/y^ slice is indicated by gray and red bars, respectively. **(F)** Uncoupling-mediated increases in Rh123 fluorescence indicating massive mitochondrial depolarization. Untreated *Mecp2*^-^^/y^ slices showed less pronounced Rh123 increases upon FCCP treatment (5 μM, 5 min), suggesting a less negative Δψ_m_ Trolox dampened this genotypic difference. Data were analyzed in CA1 *st. radiatum*. **(G)** Summary of the CN**^-^-evoked increases in Rh123 fluorescence. Plotted data are normalized to the respective effects of FCCP, i.e., the maximum depolarization induced at the end of the experiment in each slice.

To decide whether Trolox also modulates mitochondrial function, we assessed mitochondrial metabolism by imaging FAD and NADH autofluorescence (Duchen and Biscoe, 1992; Hepp et al., 2005; Foster et al., 2006), and mitochondrial membrane potential (Δψ_m_) by recording Rh123 fluorescence (Emaus et al., 1986; Duchen, 1999). These imaging experiments were performed on the tissue level in *st. radiatum* of the CA1 subfield (see **Figure 4D**), as also synaptic function was analyzed in this very layer.

As we reported earlier, the ratio of FAD/NADH autofluorescence is slightly increased (i.e., more oxidized) in *Mecp2*^-^^/y^ hippocampus, indicating an intensified basal mitochondrial respiration (Groβer et al., 2012). Also in the current experiments, the FAD/NADH ratio was increased slightly by an average of 10.9% in *Mecp2*^-^^/y^ slices (*n* = 17, *P* = 0.011) as compared to WT (*n* = 19; **Figures 4A,B**). Trolox (1 mM, 3–5 h) did not mediate any statistically significant changes in these metabolic parameters of either WT or *Mecp2*^-^^/y^ slices; the observed fading of the moderate genotypic differences in FAD/NADH ratio (**Figure 4B**) therefore seems to arise from data variability rather than a defined effect of Trolox.

In addition to basal metabolism we also defined the impact of pharmacological inhibition of the respiratory chain. Application of low and high doses of CN**^-^ (100 μM, 1 mM), caused the expected dose-dependent decreases in FAD/NADH ratio (up to -29.9 ± 2.5%, *n* = 19 in WT; up to -30.8 ± 2.6%, *n* = 17 in *Mecp2*^-^**^/y^), which were indistinguishable among untreated slices (**Figures 4A,C**). Trolox (1 mM, 3–5 h) slightly but significantly dampened the inhibitory effects of both low (*P* = 0.008) and high (*P* = 0.021) CN^-^ concentrations in *Mecp2*^-^**^/y^ slices, by an average of 13.5 and 10.1%, respectively (*n* = 19, **Figure 4C**), suggesting that it may reduce the susceptibility of mitochondrial metabolism against (chemical) anoxia. In WT slices, such dampening effects of Trolox did not occur (*n* = 15).

Our earlier experiments also suggested a partly depolarized Δψ_m_ in *Mecp2*^-^**^/y^ hippocampus (Groβer et al., 2012). Performing corresponding experiments confirmed less intense Δψ_m_ responses in *Mecp2*^-^**^/y^ slices (*n* = 20, *P* = 0.030) upon mitochondrial uncoupling by 5 μM FCCP than in WT (*n* = 20; **Figures 4E,F**), and hence a less negative Δψ_m_. Upon Trolox treatment, both WT (*n* = 15) and *Mecp2*^-^**^/y^ slices (*n* = 17) tended to show slightly more intense Rh123 responses to FCCP; as a result, the genotypic difference became smaller and was no longer statistically significant (**Figure 4F**).

Cyanide-mediated inhibition of mitochondrial respiration evoked marked increases in Rh123 fluorescence, indicating strong mitochondrial depolarization (**Figures 4E,G**). For better comparability, these Rh123 changes were normalized to the complete mitochondrial depolarization induced by FCCP. Genotypic differences were, however, not observed in the responses of WT and *Mecp2*^-^^/y^ slices to low and high CN**^-^ doses (*n* = 20 each; **Figure 4G**). Neither did Trolox significantly modulate the extent of the CN**^-^-induced mitochondrial depolarization. Only in the case of high CN**^-^ concentrations, the Rh123 responses tended to be slightly higher in Trolox-treated *Mecp2*^-^^/y^ slices (*n* = 17, *P* = 0.059).

## DISCUSSION

Presently, there is no cure for RS, but a number of pharmacotherapeutic strategies ameliorate certain aspects of the complex clinical presentation [see: (Chapleau et al., 2013)]. Some of these treatments aim to prevent oxidative stress by improving cellular redox balance. Curcumine-feeding of female Rett mice dampens the intensified ROS generation in mesenteric artery and reinstates normal vasculature function (Panighini et al., 2013). Initial verification of antioxidant treatment in Rett patients confirms that oral supplementation with ω-3 polyunsaturated fatty acids successfully decreases the clinical severity score by improving motor function, non-verbal communication and breathing (De Felice et al., 2012a).

Here we analyzed to what degree the radical scavenger Trolox ameliorates neuronal function in the MeCP2-deficient mouse hippocampal network. Our choice of a vitamin E derivative was based on the high scavenging efficiency of this class of compounds, and Trolox in particular was selected due to its water solubility. Vitamin E derivatives outrun any destructive interactions of hydroperoxyl radicals with polyunsaturated fatty acids by scavenging these radicals at ~1.000-fold faster kinetics and thereby break lipid peroxidation chain reactions (Buettner, 1993; Traber and Stevens, 2011; Alberto et al., 2013). Furthermore, they also react with singlet oxygen as well as superoxide, and decrease the cell endogenous H_2_O_2_ formation (Brigelius-Flohé and Traber, 1999; Peus et al., 2001). Most importantly, vitamin E is not degraded in the scavenging process but is rather “recycled” to its reduced state by, e.g., vitamin C (Buettner, 1993; Traber and Stevens, 2011), which further optimizes its efficiency.

Indeed, in isolated tissue of already symptomatic Rett mice, acute 3–5 h Trolox treatment clearly dampened neuronal hyperexcitability, improved synaptic plasticity, and increased the tolerance to severe hypoxia. As shown previously this free radical scavenger also decreases the elevated (more oxidized) redox baselines in *Mecp2*^-^^/y^ hippocampal slice cultures and dampens the exaggerated redox responses to oxidant challenge (Groβer et al., 2012). Less clear effects of Trolox were, however, observed on mitochondria and seizure-like activity.

In detail, in the hippocampal network, Trolox improved basal synaptic function by selectively dampening neuronal hyperexcitability in* Mecp2*^-^^/y^ but not in WT slices. As a result, the normalized fEPSPs reached amplitudes typical of untreated WT slices. Mechanistically, a modulation of neuronal network function by changes in cellular redox balance is difficult to predict, since various pivotal ion-channels and transmitter receptors are modulated to different degrees and may even respond oppositely. For example oxidant challenge blocks NMDA and GABA_A_ receptors (Aizenman et al., 1989; Sah et al., 2002) but activates voltage-gated Na^+^ channels and ryanodine receptors (Hammarström and Gage, 2000; Hidalgo et al., 2004; Gerich et al., 2009). Since we did not perform detailed pharmacological trials, the molecular origin of hyperexcitability in *Mecp2*^-^^/y^ mice is unclear. Yet, independent of its mechanism, the normalization of excitability by Trolox may well be of importance in view of the pronounced seizure susceptibility associated with RS and it may also contribute to the postponed onset of HSD in Trolox-treated *Mecp2*^-^^/y^ slices.

Trolox also improved various aspects of synaptic plasticity which is an important finding in view of the severe cognitive impairment in RS. PPF was not primarily affected, but the genotypic differences among WT and *Mecp2*^-^^/y^ slices under control conditions, were no longer present upon Trolox treatment. More importantly the extent of STP was improved by Trolox and LTP was fully restored to its normal extent. As especially long-term plasticity improved, it seems that in particular postsynaptic structures were modulated by the radical scavenger treatment. LTP induction at Schaffer collateral/CA1 synapses is NMDA-receptor dependent (Bliss and Collingridge, 1993). It is therefore tempting to speculate that the more oxidizing conditions in *Mecp2*^-^^/y^ hippocampus partially inactivate the oxidation-sensitive NMDA receptors (Aizenman et al., 1989) and thus contribute to the less stable LTP seen in Rett mouse hippocampus (Asaka et al., 2006; Moretti et al., 2006; Guy et al., 2007). Along this line, the Trolox-mediated normalization of redox balance may have restored normal NMDA receptor function and thus LTP.

In WT slices, however, the extent of STP was dampened by Trolox and also LTP tended to be depressed. In this aspect the modulation of synaptic plasticity by Trolox differs from its effects on basal synaptic function, where no effects on WT were observed. A reasonable explanation for these findings is the strict dependence of LTP on exact cellular redox balance. Oxidant stress may interfere with LTP maintenance without affecting STP or PPF (Pellmar et al., 1991). Yet, also reducing shifts due to overexpression of extracellular SOD3 or administration of superoxide scavengers impair hippocampal LTP (Klann et al., 1998; Thiels et al., 2000). It therefore seems that ROS do not only oppose the induction of stable LTP but to some degree are essential for synaptic plasticity (Massaad and Klann, 2011). This emphasizes the importance of a well-balanced cellular redox equilibrium and hence optimized dosage of redox-modulators such as radical scavengers. We tested only a single concentration of Trolox (1 mM), and observed improved LTP in *Mecp2*^-^^/y^ slices, but its partial depression in WT. Accordingly, a more careful titration of redox conditions may be required to ensure that LTP improves in *Mecp2*^-^^/y^ slices without dampening synaptic plasticity in WT.

Despite a selective dampening of neuronal excitability in *Mecp2*^-^^/y^ slices by Trolox, we did not observe a marked reduction in seizure susceptibility. Only in WT, the onset of SLEs tended to be postponed and the duration of the individual SLEs only tended to be decreased by Trolox in both WT and *Mecp2*^-^^/y^ slices. Even though the latter occurred in both genotypes, it may be of some profit by dampening the severity of seizures once such abnormal discharges are triggered. It should be considered, however, that the K^+^ channel inhibitor 4-AP is a rather strong convulsive stimulus. Nevertheless, as a pronounced seizure susceptibility is associated with RS, and even constitutes a potential cause for sudden death (Hagberg et al., 1983; Steffenburg et al., 2001), it is an important finding that the Trolox-mediated normalization of synaptic plasticity in *Mecp2*^-^^/y^ hippocampus is not associated with negative side effects such as increased neuronal excitability and/or increased seizure susceptibility.

Trolox also abolished the increased susceptibility of *Mecp2*^-^^/y^ hippocampus to the lack of O_2_. The hastened onset of HSD in *Mecp2*^-^^/y^ slices was selectively reverted to WT conditions, whereas WT slices were not affected. Hence, the normalized hypoxia susceptibility constitutes another protective effect that was induced by Trolox selectively in *Mecp2*^-^^/y^ slices. Treatments decreasing neuronal excitability postpone the onset of spreading depression while increased excitability favors its occurrence [see (Somjen, 2001)]. Therefore, the postponement of HSD in *Mecp2*^-^^/y^ slices by Trolox is very likely a result of the observed selective dampening of neuronal excitability, i.e., fEPSP/fiber volley ratios. In contrast, Trolox-treated WT slices did not show any alterations in neuronal excitability nor HSD onset. Also increased ROS levels (Grinberg et al., 2012), changes in thiol redox balance (Hepp et al., 2005; Hepp and Müller, 2008), and mitochondrial inhibition (Gerich et al., 2006) modulate the induction threshold of spreading depression. Hence, the postponement of HSD in Trolox-treated *Mecp2*^-^^/y^ slices may also partly be due to the stabilized redox balance or a slightly improved mitochondrial anoxia tolerance that is suggested by the milder CN**^-^ effects in Trolox-treated *Mecp2*^-^^/y^ slices. In view of the highly irregular breathing and the associated intermittent systemic hypoxia in RS (Julu et al., 2001; Stettner et al., 2008; Katz et al., 2009), the Trolox-mediated increase in hypoxia tolerance is clearly of potential merit, as it may prevent additional complications especially in anoxia vulnerable neuronal networks such as the hippocampus and cortex.

Mitochondria are a primary cellular source of ROS (Boveris and Chance, 1973; Adam-Vizi, 2005), and mitochondrial alterations in RS underlie the increased oxidative burden and altered cellular redox homeostasis (Groβer et al., 2012). However, mitochondria are also potential targets for oxidative damage, and respond with morphological and functional changes including altered intracellular trafficking (Petronilli et al., 1994; Gerich et al., 2009; Qi et al., 2011; Lenaz and Genova, 2012). We therefore screened whether Trolox may modulate mitochondrial function directly, but a noticeable improvement of mitochondrial function was not observed. The genotypic baseline differences in FAD/NADH ratio were not significantly affected by Trolox. During 1 mM CN**^-^ treatment, it mediated only a moderate dampening effect on the anoxic drop in FAD/NADH ratio, which may suggest an increased anoxia tolerance of mitochondrial respiration, yet any corresponding effects on Δψ_m_ were not observed. Also the trend to slightly increased Rh123 responses in both WT and *Mecp2*^-^^/y^ slices during uncoupling or the tendency of somewhat increased Rh123 responses to high CN**^-^ concentrations in *Mecp2*^-^^/y^ slices may suggest some improvement of Δψ_m__,_ but as stated, the level of significance was not reached. It therefore seems that the protective effects of vitamin E reported for rat liver mitochondria, i.e., partial normalization of the increased state 3 and state 4 respiration upon acute lipid peroxidation (Ham and Liebler, 1997), do not apply to hippocampal mitochondria and the chronic oxidative stress associated with RS. Yet, at least our data confirm that Trolox does not mediate any adverse side effects on mitochondrial function in WT and especially in *Mecp2*^-^^/y^ slices.

## CONCLUSION

The free radical scavenger treatment performed in our study verifies the potential merit of Trolox for targeting the aberrant redox conditions that manifest in MeCP2-deficient networks. In isolated *Mecp2*^-^^/y^ hippocampal tissue of symptomatic mice we confirmed an improvement of various aspects of neuronal network function, including synaptic plasticity, neuronal excitability, and hypoxia tolerance. At the same time we ruled out potentially adverse side effects on mitochondrial metabolism and seizure susceptibility.

Of course, the hippocampus shows a tight coupling of neural function, metabolism and cellular redox balance, as it is highly vulnerable (Schmidt-Kastner and Freund, 1991), exhibits a clear basal ROS production (Bindokas et al., 1996) and its CA1 neurons are particularly sensitive to oxidative stress (Wilde et al., 1997; Wang et al., 2007). Therefore, Trolox now should undergo further detailed tests to clarify, how other, less vulnerable brain areas respond to radical scavengers and/or modulation of cellular redox homeostasis. This should also include more complex preparations up to *in vivo* treatment of Rett mice to carefully define the merits but also limitations of these compounds. In RS mitochondrial dysfunction and redox imbalance manifest early in life, and may contribute to disease progression. It is therefore crucial to start radical scavenger treatment at presymptomatic stages to define potential changes in disease progression and the manifestation of typical symptoms. Only then, these compounds may unveil their full pharmacotherapeutic potential for the treatment of RS.

## Conflict of Interest Statement

The authors declare that the research was conducted in the absence of any commercial or financial relationships that could be construed as a potential conflict of interest.
